# Serum D-dimer level as a biomarker for identifying patients with isolated injury to prevent unnecessary whole-body computed tomography in blunt trauma care

**DOI:** 10.1186/s13049-020-00815-9

**Published:** 2021-01-07

**Authors:** Rakuhei Nakama, Ryo Yamamoto, Yoshimitsu Izawa, Keiichi Tanimura, Takashi Mato

**Affiliations:** 1 Department of Emergency Medicine, Saiseikai Utsunomiya Hospital, 911-1 Takebayashi-machi, Utsunomiya, Tochigi 321-0974 Japan; 2 Department of Emergency and Critical Care Medicine, Jichi Medical University, 3311-1 Yakushiji, Shimotsuke, Tochigi 329-0498 Japan; 3 Department of Emergency and Critical Care Medicine, Keio University school of medicine, 35 Shinanomachi, Shinjuku, Tokyo, 160-8582 Japan; 4 Department of Radiology, Saiseikai Utsunomiya Hospital, 911-1 Takebayashi-machi, Utsunomiya, Tochigi 321-0974 Japan

**Keywords:** Computed tomography, Whole-body CT, D-dimer, Blunt trauma, Multiple trauma, Radiation exposure

## Abstract

**Background:**

Unnecessary whole-body computed tomography (CT) may lead to excess radiation exposure. Serum D-dimer levels have been reported to correlate with injury severity. We examined the predictive value of serum D-dimer level for identifying patients with isolated injury that can be diagnosed with selected-region CT rather than whole-body CT.

**Methods:**

This single-center retrospective cohort study included patients with blunt trauma (2014–2017). We included patients whose serum D-dimer levels were measured before they underwent whole-body CT. “Isolated” injury was defined as injury with Abbreviated Injury Scale (AIS) score ≤ 5 to any of five regions of interest or with AIS score ≤ 1 to other regions, as revealed by a CT scan. A receiver operating characteristic curve (ROC) was drawn for D-dimer levels corresponding to isolated injury; the area under the ROC (AUROC) was evaluated. Sensitivity, specificity, positive predictive value, and negative predictive value were calculated for several candidate cut-off values for serum D-dimer levels.

**Results:**

Isolated injury was detected in 212 patients. AUROC was 0.861 (95% confidence interval [CI]: 0.815–0.907) for isolated injury prediction. Serum D-dimer level ≤ 2.5 μg/mL was an optimal cutoff value for predicting isolated injury with high specificity (100.0%) and positive predictive value (100.0%). Approximately 30% of patients had serum D-dimer levels below this cutoff value.

**Conclusion:**

D-dimer level ≤ 2.5 μg/mL had high specificity and high positive predictive value in cases of isolated injury, which could be diagnosed with selected-region CT, reducing exposure to radiation associated with whole-body CT.

## Background

Computed tomography (CT) is widely used to evaluate patients with traumatic injuries [1]. Physical assessment typically determines whether CT is necessary and the region where it needs to be performed; however, whole-body CT rather than selected-region CT is often performed without obvious indications such as disturbance of consciousness or presence of distracting painful injury [2]. In contrast to selected-region CT, whole-body CT can help to promptly detect multiple injuries that require immediate intervention [3, 4]; nevertheless, indications for whole-body CT remain undefined [5].

Unnecessary whole-body CT may lead to excess radiation exposure. Whole-body CT exposes large body surface areas to radiation; when contrast imaging is performed, the total amount of radiation delivered to the patient can be more than three-fold greater than that delivered during select-region CT [6]. Given the variation in sensitivity to radiation, guidelines recommend careful use of whole-body CT in children [7].

Previous studies have suggested that candidates should be selected for whole-body CT based on trauma mechanism, which could cause severe injury at a non-obvious site. Recent studies have shown that serum D-dimer levels measured immediately after trauma correlate with patients’ injury severity scores (ISS) [8], suggesting that D-dimer levels might be a biomarker for whole-body CT suitability. Considering that emerging point-of-care devices have now enabled physicians to measure serum D-dimer within in a few minutes, the use of D-dimer levels along with careful physical examination could help to reduce the use of unnecessary whole-body CT.

This study examined an association between serum D-dimer levels and suitability of whole-body CT in trauma patients with multiple injuries. We hypothesized that low D-dimer levels are associated with isolated injury that could be detected by selected-region CT, rather than whole-body CT, in patients who are alert and hemodynamically stable.

## Methods

This was a single-center retrospective cohort study conducted at Saiseikai Utsunomiya Hospital, Tochigi, Japan. Recommendation for a whole-body CT was at the discretion of the attending physician, who considered the following indications: severe injury or unknown mechanism, altered mental status, distractingly painful injury, or multiple injuries identified or suspected at physical examination.

The protocol of whole-body CT included a non-contrast CT from the head to the pelvis, an arterial phase from the neck to the pelvis, and a venous phase from the neck to the pelvis. When a limb injury was suspected, the relevant extremity was added to the range of whole-body CT. A 64 detector CT scanner (SOMATOM Definition AS, (Siemens Healthcare, Erlangen, Germany) was used during the study period.

### Study population

We extracted data on patients who sustained blunt trauma injuries between January 1, 2014 and April 30, 2017. We included patients whose serum D-dimer levels were measured before they underwent whole-body CT with contrast, within 24 h after injury. We excluded patients with systolic blood pressure (sBP) < 90 mmHg and those with Glasgow Coma Scale (GCS) score < 15 at admission. We also excluded patients with any neurological abnormalities, indicative of injuries that could not be diagnosed using CT, including spinal cord and peripheral nerve injury. Patients who underwent surgery or angiography before whole-body CT were also excluded.

### Data collection and definition

Data were extracted from electronic medical records, including information on age, gender, mechanism of injury, vital signs at admission (GCS score, respiratory rate, sBP, and heart rate), abbreviated injury scale (AIS) score, ISS score, Revised Trauma Score, Trauma and Injury Severity Score Probability of Survival, serum D-dimer level, and detailed information obtained from whole-body CT. All images acquired through whole-body CT were re-evaluated by board-certified radiologists not otherwise involved in this study. Disagreements between radiologists were resolved by discussion.

Injury sites were divided into five regions (head/neck, face, chest, abdomen, and limbs/pelvis) according to the AIS coding system. “Isolated” injury was defined as an AIS score ≤ 5 in one of five regions, and an AIS score ≤ 1 in one of other four regions. “Isolated non-severe” injury was defined as an AIS score ≤ 3 in one of five regions and an AIS score ≤ 1 in one of four regions.

### Outcome measures

Primary outcome was defined as isolated injury; secondary outcome was defined as isolated non-severe injury.

### Statistical analysis

The suitability of using serum D-dimer levels to predict primary and secondary outcomes was assessed by discrimination and reclassification analysis. Receiver operating characteristic (ROC) curves for D-dimer levels, according to isolated injury status, were drawn; the area under the ROC curve (AUROC) was evaluated. Sensitivity, specificity, positive predictive value (PPV), and negative predictive value (NPV) were calculated for several candidate D-dimer level cutoff values to obtain the lowest possible value that was most likely to eliminate the need for whole-body CT (i.e., a value that predicted an isolated injury).

Sensitivity analyses were performed on a validation cohort of patients with GCS score of 13–14 points at admission, added to the original study population to assess the robustness of the proposed model. Same statistical analyses were applied in sensitivity analyses.

All statistical analyses were performed using EZR version 1.42 (Saitama Medical Center, Jichi Medical University, Saitama, Japan) [9], which is a graphical user interface for R (The R Foundation for Statistical Computing, Vienna, Austria).

## Results

**Fig. 1:**
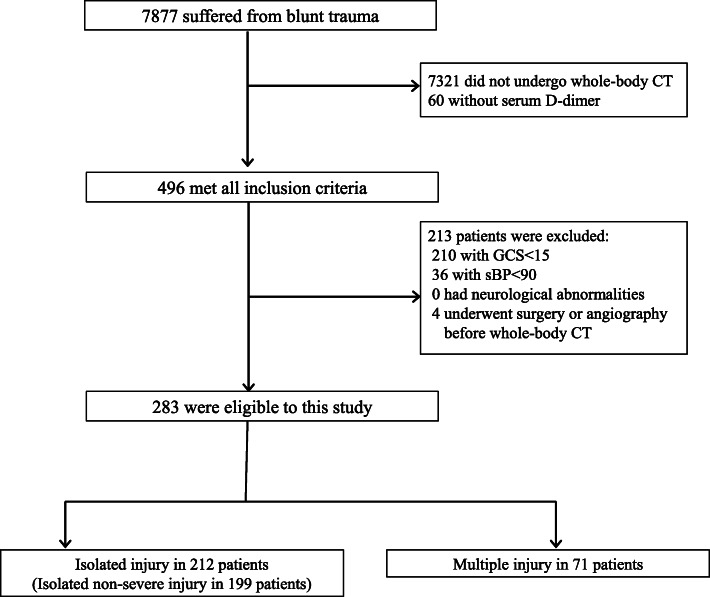
Patient eligibility flow chart, including the number of patients included and excluded from the study, with reasons

A total of 7877 patients with blunt trauma were identified during the study period (Fig. 1). Of these, 7321 patients did not undergo whole-body CT, and 60 patients did not have serum D-dimer values. A total of 496 patients met all the inclusion criteria, of whom, 210 and 36 patients were excluded due to GCS scores < 15 and sBP < 90, respectively. Four patients were also excluded because surgery or angiography was performed before whole-body CT. No patient was excluded due to neurological abnormalities. Accordingly, 283 patients were eligible for inclusion in this study.

The patients’ median age was 47 years; most patients (70.7%) were men. Mechanism of injury was comprised traffic accident (73.9%) and fall (19.1%). The main injury sites (AIS score ≥2) were the pelvis/extremity (27.9%) and chest/thoracic spine (25.1%). The median ISS, RTS, Ps were 4, 7.841, and 99.1%, respectively. The median D-dimer level was approximately 7.2 μg/mL, and the numbers of patients within each D-dimer level range were as follows: < 0.5 μg/mL, 4 (1.4%); 0.5–1.0 μg/mL, 27 (9.5%); 1.0–3.0 μg/mL, 53 (18.7%); 3.0–5.0 μg/mL, 35 (12.4%); and ≥5.0 μg/mL, 164 (58.0%). Isolated injury was detected in 212 patients, and isolated non-severe injury was detected in 199 patients (Table 1).

**Table 1:** Baseline demographic and clinical characteristics of trauma patients

Characteristic	Number of patients (***n***=283)
Age, years, median (IQR)	47 (30–64)
Sex, male, n (%)	200 (70.7%)
Mechanism of injury, n (%)	
Motor vehicle collision	209 (73.9%)
Fall	57 (20.1%)
Other	17 (6.0%)
Injury site^a^, n (%)	
Head and neck	46 (16.3)
Face	2 (0.7)
Chest	71 (25.1)
Abdomen	49 (17.3)
Pelvis/Extremity	79 (27.9)
Vital signs on presentation	
GCS	15 (15–15)
Respiratory rate, /min	21 (17–25)
Systolic blood pressure, mmHg	140 (123–161)
Heart rate, /min	81 (72–94)
D-dimer, μg/mL, median (IQR)	7.2 (2.3–23.7)
< 0.5 μg/mL, n (%)	4 (1.4%)
0.5–1.0 μg/mL, n (%)	27 (9.5%)
1.0–3.0 μg/mL, n (%)	53 (18.7%)
3.0–5.0 μg/mL, n (%)	35 (12.4%)
≥5.0, n (%)	164 (58.0%)
Injury Severity Score, median (IQR)	4 (0–13)
Revised Trauma Score, median (IQR)	7.84 (7.84–7.84)
Probability of survival, median (IQR)	0.99 (0.98–1.00)
Isolated injury, n (%)	212 (74.9%)
Insolated non-severe injury, n (%)	199 (70.3%)

*GCS* Glasgow Coma Scale, ^a^includes injuries with AIS score ≥2

### Discrimination and reclassification power of the model

**Fig. 2:**
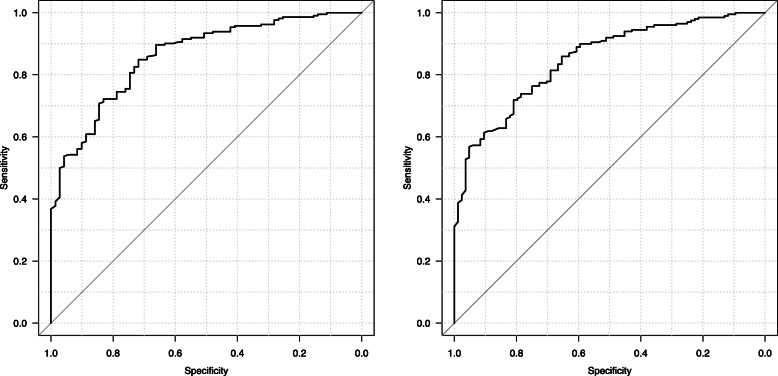
Receiver operating characteristic curves for D-dimer levels according to the isolated injury (**a**) and isolated non-severe injury (**b**)

The ROC curves for D-dimer levels corresponding to isolated and isolated non-severe injury are shown in Fig. 2. The AUROC was 0.861 (95% confidence interval [CI]: 0.815–0.907) for isolated injury and 0.849 (95% CI: 0.804–0.894) for isolated non-severe injury. Sensitivity analyses on the validation cohort revealed a similar discrimination power of D-dimer levels, with corresponding AUROC values of 0.859 (95% CI: 0.819–0.895) and 0.849 (95% CI: 0.804–0.894).

A cutoff value of 2.5 μg/mL had a high specificity (100.0%) and PPV (100.0%) for predicting isolated injury; approximately 30% (76/283) of patients had serum D-dimer levels below this value (Table 2). Moreover, the cutoff value of 2.5 μg/mL had a high specificity (98.8%) and PPV (98.7%) for predicting isolated non-severe injury. In sensitivity analyses, cutoff value ≤ 2.5 μg/mL had a high specificity (98.3%) and PPV (98.0%) for predicting isolated injury in the validation cohort that included patients with sBP ≥90 mmHg and GCS score ≥13 at admission (Table 3).

**Table 2:** Serum D-dimer levels associated with isolated injury

Cut-off value of D-dimer (μg/mL)	≤0.5	≤1.0	≤2.0	≤2.5	≤5.0	≤10.0	≤20.0
Sensitivity (%)	3.3	17.0	31.1	35.8	54.2	71.7	86.8
Specificity (%)	100.0	100.0	100.0	100.0	93.0	83.1	66.2
Positive predictive value (%)	100.0	100.0	100.0	100.0	95.1	92.7	88.5
Negative predictive value (%)	25.7	29.0	33.0	34.3	41.1	50.0	63.2
Number of patients with D-dimer ≤ cut-off value, n	7	36	66	76	120	164	207
Proportion of patients with D-dimer ≤ cut-off value, %	2.5	12.7	23.3	26.9	42.4	58.0	73.1

**Table 3:** Predictive ability of D-dimer levels associated with isolated non-severe injury and isolated injury in extended population

D-dimer ≤2.5 μg/mL	Isolated non-severe injury	Isolated injury in extended population
Sensitivity (%)	37.7	35.4
Specificity (%)	98.8	98.3
Positive predictive value (%)	98.7	98.0
Negative predictive value (%)	40.1	39.9

## Discussion

This study identified serum D-dimer level ≤ 2.5 μg/mL as predictive of isolated injury that could be detected with selected-region rather than whole-body CT; the model had a high specificity and high PPV. Similarly, the same cutoff value for D-dimer level had > 95% specificity and PPV for isolated non-severe injury. In this study, almost one-third of the patients had serum D-dimer levels below this cutoff, suggesting that using this threshold could spare a considerable number of patients from undergoing whole-body CT.

Serum D-dimer level measured immediately after trauma has been previously suggested as a marker indicative of injury severity. A recent retrospective study reported that serum D-dimer level was associated with injury severity and unfavorable clinical outcomes in trauma patients [8, 10, 11]. Moreover, other studies have shown that serum D-dimer levels were associated with the number of fractures and mild traumatic brain injury detected by CT [12, 13]. In addition, pathophysiological studies have found that endothelial damage due to blunt trauma triggered coagulation and enhanced fibrinolysis, resulting in elevated serum D-dimer levels [14]. Furthermore, it has been suggested that tissue hypoperfusion caused by injuries leads to the acute release of t-PA from endothelial cells; thus, the degree of increment in serum D-dimer level would be related to the extent of injured sites [15]. In the present study, D-dimer level was indicative of isolated injury, suggesting its suitability as a candidate marker for determining the necessity of whole-body CT.

Exposure to radiation associated with whole-body CT is a concern in trauma care worldwide. A recent retrospective study aimed to develop a prediction model that could reduce the number of unnecessary whole-body CT scans among trauma patients [16]. Although the proposed model had a high sensitivity for multiple injuries with AIS score > 1 or single injuries with AIS score > 2, it required the input of several other variables, including injury mechanism, number of injury sites, and details of vital signs. Meanwhile, a prospective observational study concluded that physician judgement based on patient history and/or physical examination, including vital signs, is insufficient to determine the necessity of hole-body CT [17]. In contrast, the model presented in the present study is based solely on serum D-dimer levels that predict isolated injury, which can be confirmed by selected-region CT; this model can be easily applied in trauma centers worldwide. Notably, point-of-care tests for serum D-dimer levels have been developed and are available to physicians; using these tests, D-dimer levels can be determined within 10 min after hospital arrival [18].

The specificity of serum D-dimer level cutoff value presented in this study is similar to that in other validated screening tests used in emergency settings, such as rapid influenza virus antigen test or troponin T test for myocardial infarction [19, 20]. These tests, which have 98% specificity, have been used as reliable qualitative indicators in urgent care, suggesting that serum D-dimer levels ≤ 2.5 μg/mL, with a similar specificity value, might be suitable for use in an emergency trauma setting. Finally, as approximately 30% of included patients satisfied this cutoff value, it is likely a useful parameter in the treatment of blunt trauma patients.

This study has some limitations, which should be considered when interpreting its findings. Although D-dimer level has a high specificity and high positive predictive value for isolated injury, the presented cutoff value was not validated with data from an independent cohort. Differences in study settings, including regional trauma system, trauma evaluation system at hospitals, and patient characteristics, likely limit the generalizability of our findings. Moreover, the proposed D-dimer level cutoff value can only help to exclude whole-body CT from the diagnostic process; however, it is not indicative of the regions that should targeted with selected-region CT, which may require vital sign analysis or physical examination.

Another limitation is that we included only patients in whom the attending physicians had decided to conduct whole-body CT based on the clinical information. Therefore, the D-dimer would not be useful in situations where the attending physicians can rule out the necessity of whole-body CT based on the history and/or physical examination. Furthermore, it should be emphasized that preexisting stringent institutional trauma protocols that indicate the candidates for whole-body CT would prevent the D-dimer screening from being adopted.

Finally, due to the retrospective nature of this study, the presented findings are not conclusive. Unmeasured confounding factors, including comorbidities such as pulmonary embolism and deep venous thrombosis, can increase D-dimer levels, affecting the precision of the proposed model [21, 22]. Furthermore, patients in whom the blood sample is drawn too early (e.g. on the scene) or those who have coagulative diseases as comorbidities may have had low D-dimer levels (≤2.5 μg/mL) which could contribute to the false negative results. Prospective studies are required to evaluate the utility and predictive value of the proposed indicator.

## Conclusions

Serum D-dimer level ≤ 2.5 μg/mL has a high specificity and high PPV for predicting isolated injury that could be diagnosed with selected-region rather than whole-body CT. These findings can help to reduce the number of unnecessary whole-body CT scans performed in trauma care.

## Data Availability

Not applicable.

## Abbreviations

AIS: Abbreviated injury scale
AUROC: Area under the receiver operating characteristic curve
CT: Computed tomography
GCS: Glasgow Coma Scale
ISS: Injury severity score
NPV: Negative predictive value
PPV: Positive predictive value
ROC: Receiver operating characteristic curve
sBP: Systolic blood pressure
